# 16S rRNA Sequencing and Metagenomics Study of Gut Microbiota: Implications of BDB on Type 2 Diabetes Mellitus

**DOI:** 10.3390/md18090469

**Published:** 2020-09-17

**Authors:** Liang Zhang, Jiao Luo, Xiangqian Li, Shuju Guo, Dayong Shi

**Affiliations:** 1 Key Laboratory of Experimental Marine Biology, Institute of Oceanology, Chinese Academy of Sciences, 7 Nanhai Road, Qingdao 266071, China; zl1172627930@163.com; 2 Laboratory for Marine Drugs and Bioproducts of Qingdao, National Laboratory for Marine Science and Technology, Qingdao 266000, China; 3 School of Earth and Planetary, University of Chinese Academy of Sciences, Beijing 100049, China; 4 School of Public Health, Qingdao University, Qingdao 266071, China; luojiao2012@163.com; 5 State Key Laboratory of Microbial Technology, Shandong University, 72 Binhai Road, Qingdao 266237, China; lixiangqian@sdu.edu.cn

**Keywords:** BDB, marine red alga, type 2 diabetes mellitus, gut microbiota, 16S rRNA sequencing, metagenomics

## Abstract

Gut microbiota has a critical role in metabolic diseases, including type 2 diabetes mellitus (T2DM). 3-bromo-4,5-bis(2,3-dibromo-4,5-dihydroxybenzyl)-1,2-benzenediol (BDB) is a natural bromophenol isolated from marine red alga *Rhodomela confervoides*. Our latest research showed that BDB could alleviate T2DM in diabetic BKS db mice. To find out whether BDB modulates the composition of the gut microbiota during T2DM treatment, 24 BKS db diabetic mice were randomly grouped to receive BDB (*n* = 6), metformin *(n* = 6), or the vehicle (*n* = 6) for 7 weeks in a blinded manner. Non-diabetic BKS mice (*n* = 6) were used as normal control. Diabetic mice treated with BDB or metformin demonstrated significant reductions in fasting blood glucose (FBG) levels compared with the vehicle-treated mice in the 7th week. Pyrosequencing of the V3–V4 regions of the 16S rRNA gene revealed the changes of gut microbiota in response to BDB treatment. The result demonstrated short-chain acid (SCFA) producing bacteria Lachnospiraceae and Bacteroides were found to be significantly more abundant in the BDB and metformin treated group than the vehicle-treatment diabetic group. Remarkably, at the genus levels, *Akkermansia* elevated significantly in the BDB-treatment group. Metagenomic results indicated that BDB may alleviate the metabolic disorder of diabetic mice by promoting propanoate metabolism and inhibiting starch and sucrose metabolism, amino sugar and nucleotide sugar metabolism. In conclusion, our study suggests that the anti-diabetic effect of BDB is closely related to the modulating structure of gut microbiota and the improvement of functional metabolism genes of intestinal microorganisms.

## 1. Introduction

Type 2 diabetes mellitus (T2DM), which is the most common type of diabetes, is characterized by insulin resistance, insulin deficiency, or β-cell failure [1,2]. The development and occurrence of T2DM are mostly related to obesity, family history, and diet [3]. It has become increasingly prevalent worldwide [4]. The estimated number of diabetics will reach about 700 million in 2045, based on the 9th edition of Diabetes Atlas released by the International Diabetes Federation. Hence, it has become urgent to change the present situation.

Now, the treatment of diabetes is mainly through oral hypoglycemic drugs. The Food and Drug Administration(FDA) has approved 12 kinds of drugs for controlling blood sugar in patients, including sulfonylureas, meglitinides, biguanide, α-glucosidase, and dipeptidyl peptidase(DPP-4) inhibitors, and others. However, the existing drugs for the treatment of type 2 diabetes cannot effectively control the blood sugar level for a long time in most patients, and will have some side effects [5,6]. Therefore, we urgently need to constantly develop new drugs and explore new therapeutic targets.

Recently, the main interest in the mechanisms of T2DM and its therapeutic drugs focused on the composition of intestinal microflora in humans and animals [7,8,9]. It has been confirmed that gut microbiota is closely associated with many metabolic diseases, such as diabetes and inflammatory bowel disease [10,11,12,13]. Compared with healthy controls, more opportunistic pathogens, such as *Bacteroides caccae*, *Clostridium hathewayi*, *Clostridium ramosum*, *Clostridium symbiosum*, and *Escherichia coli* (*E. coli*), were found in the T2DM patients [14]. It is reported that metformin alters the gut microbiome of individuals with treatment-naive type 2 diabetes mellitus, contributing to the therapeutic effects of the drug. Metformin enriches short-chain fatty acid-synthesizing bacteria, including *Shewanella*, *Blautia*, *Lactobacillus*, and *Akkermansia muciniphila* (*A. muciniphila*), and improved the expression of genes encoding metalloproteins by changing the composition and structure of gut microbiota to improve glucose tolerance of T2DM patients [7]. Metagenomic analysis of the fecal samples of 171 patients with diabetic and 174 healthy controls revealed that the butyrate-producing bacteria of diseased samples were increased, such as *Faecalibacterium prausnizii*, but opportunistic pathogens are reduced, including *Clostridium bolteae* [9]. The alpha species diversity and gene function of intestinal microflora in diabetic rats were significantly increased by oral administration of *A. muciniphila*. These results indicate that *A. muciniphila* can improve liver function in host animals, reduce glycolipid toxicity, reduce oxidative stress, inhibit inflammation, and normalize intestinal microflora, and thereby improve type 2 diabetes. Another study found that oral administration of *Clostridium butyricum* CGMCC0313.1 (CB0313.1) increased the proportion of butyrate-producing bacteria and prevented hyperglycemia and associated metabolic dysfunction in diabetic model mice [15]. In summary, these studies indicate that the development of obesity and diabetes may be related to dysbiotic microbiota, and restoration of gut microbiota homeostasis contributes to the improvement of these disease traits.

Marine algae have long been regarded as food or food additives [16]. Many studies have found that marine red algae of the family *Rhodomelaceae* (order Ceramiales) are an important source of bioactive natural substances, including abundant sources of phenolic compounds, especially bromophenols [17]. Some compounds isolated from this family showed significant radical scavenging activities, alpha-glucosidase inhibitory activities, and anti-inflammatory activities [18]. 3-bromo-4,5-bis(2,3-dibromo-4,5-dihydroxybenzyl)-1,2-benzenediol (BDB), a natural bromophenol isolated from marine red alga *Rhodomela confervoides*, is a potent protein-tyrosine phosphatase 1B inhibitor [19]. Our latest study showed that BDB exhibited significant hypoglycemic efficacy in spontaneous diabetic mice [20]. However, its mechanism of action is not yet fully understood. Therefore, we speculate that BDB might also act partly through the gut microbiota.

In order to verify the hypothesis that changing gut microbiota may contribute to alleviating hyperglycemia by BDB treatment, we combined high-throughput 16S rRNA gene sequencing and metagenomics study to investigate the effects of BDB on the composition and function of the gut microbiota in diabetic BKS db mice.

## 2. Results

### 2.1. Hypoglycemic Effects of BDB in Diabetic BKS db Mice

To characterize the hypoglycemic effect of BDB and whether it can improve the typical symptoms of type 2 diabetes, polydipsia, polyphagia and weight loss, the levels of blood glucose, food intake, water intake and body weight of each group were measured at the end of the experiment.

**Figure 1:**
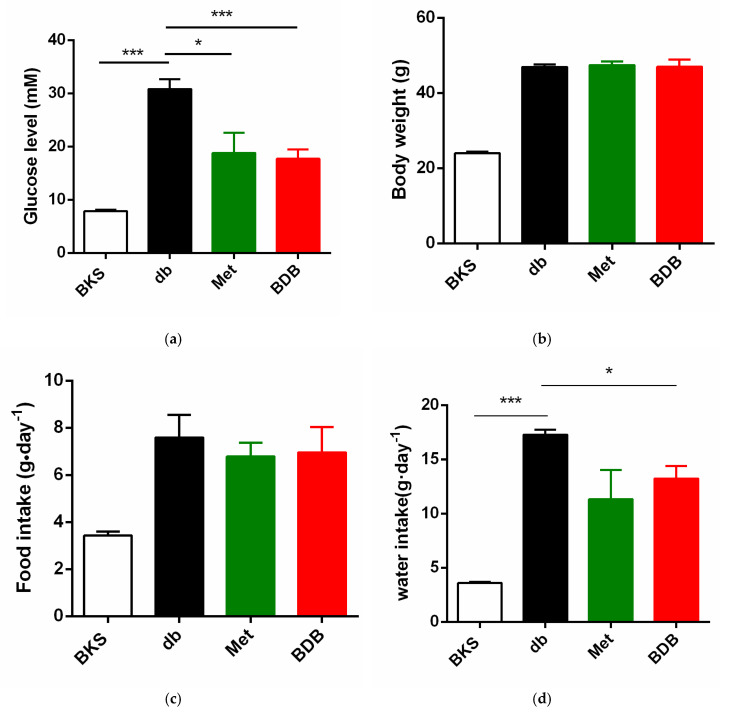
Hypoglycemic effect of 3-bromo-4,5-bis(2,3-dibromo-4,5-dihydroxybenzyl)-1,2-benzenediol (BDB). (**a**) blood glucose levels; (**b**) body weight; (**c**) food intake; (**d**) water intake in BKS db mice after 7-week oral administration with the vehicle, BDB, and Metformin, and in BKS mice treated with vehicle. Data were shown as mean ± standard error of the mean (SEM) compared with BKS db mice. * *p* < 0.05 and *** *p* < 0.001 compared with BKS db mice. db, diabetic BKS db mice; BDB, BDB-treated diabetic mice; Met, metformin-treated diabetic mice.

First, the hypoglycemic effects of BDB were verified after a 7-week oral administration of BDB in diabetic BKS db mice. As shown in Figure 1a, diabetic BKS db mice had hyperglycemia with higher blood glucose levels compared with non-diabetic BKS mice. Oral administration of BDB effectively reduced the blood glucose levels at the 7th week. The first-line T2DM drug metformin was used as the positive control. BDB had no effects on the body weight (Figure 1b) and food intake (Figure 1c), but significantly reduced the water intake of diabetic mice (Figure 1d).

### 2.2. Overall Composition of Gut Microbiota after BDB Treatment by 16S rRNA Sequencing

**Figure 2:**
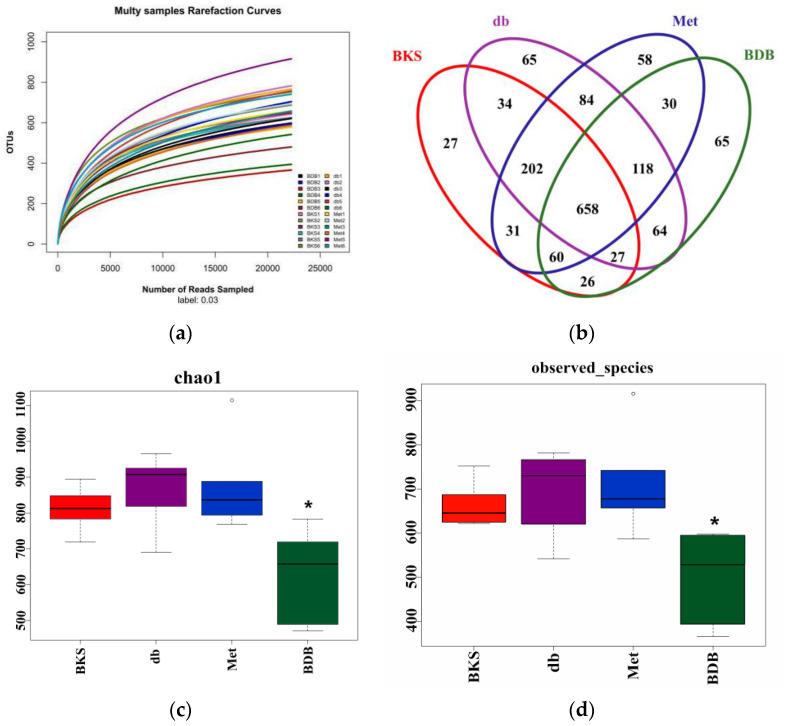
Microbial diversity and richness in mice according to different treatment. (**a**) Rarefaction curves in different treatment groups (*n* = 6); (**b**) Venn diagram showed the common and unique operational taxonomic units (OTUs) among the different group; (**c**,**d**) alpha diversity index in different treatment groups. * *p* < 0.05 compared with BKS db mice were considered statistically significant by one-way analysis of variance (ANOVA). db, diabetic BKS db mice; BDB, BDB-treated diabetic mice; Met, metformin-treated group.

16S rRNA pyrosequencing was used to analyze the overall composition of gut microbiota. We obtained a total of 1,108,806 clean tags from 1,176,921 raw tags. The rarefaction curves showed differences in microbial diversity and rationality of samples among the BKS, BKS db, metformin, and BDB groups (Figure 2a). These sequences had an average length of 400–440 base pairs, and clustered into 1549 operational taxonomic units (OTUs) at 97% 16S rRNA gene sequences identity, including 9 phyla, 92 genera, representing them as the same source of species. Six hundred and fifty-eight of the total OTUs were shared among all the samples, and some parts of them belong to unique OTUs. This showed that over 42% of the observed OTUs were not eliminated following drug treatment and that more than half of OTUs have changed, which illustrated that there were significant differences among model and BDB-treated groups (Figure 2b). According to Chao 1 and observed species richness index, the diversity of microbiota decreased significantly in the BDB-treatment group compared with other groups (*p* < 0.05), which was consistent with the previous study. Zhang et al., found berberine and metformin, significantly reduced both the richness and diversity of the gut microbiota in rats [21]. But no significant difference was observed in the metformin-treatment group compared with the diabetic model group (*p* > 0.05) (Figure 2c–d).

**Figure 3:**
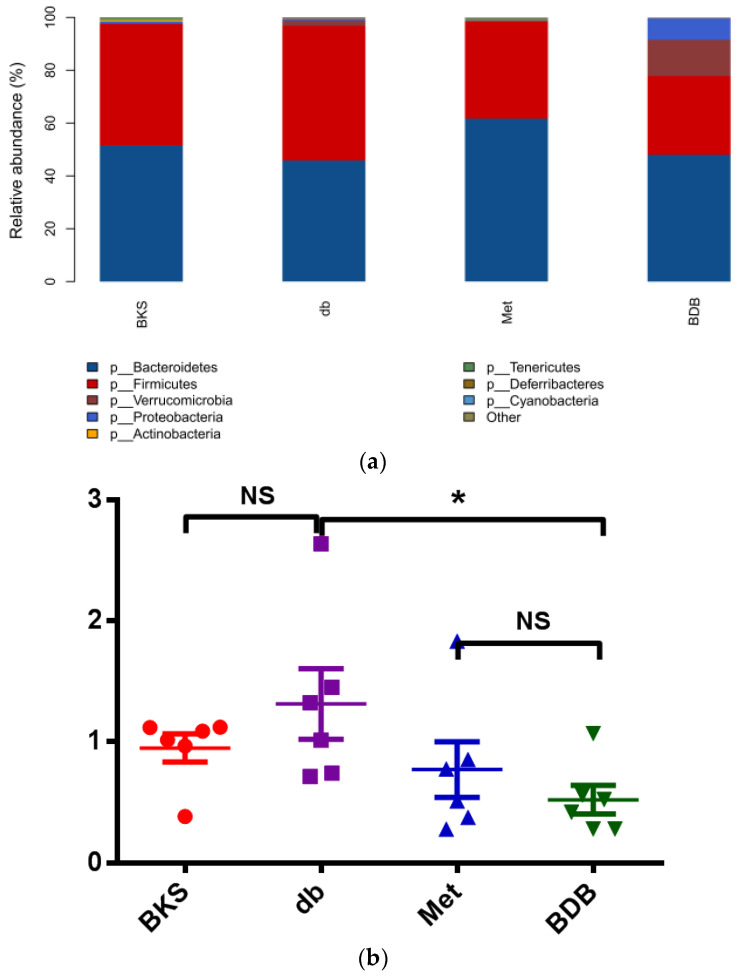

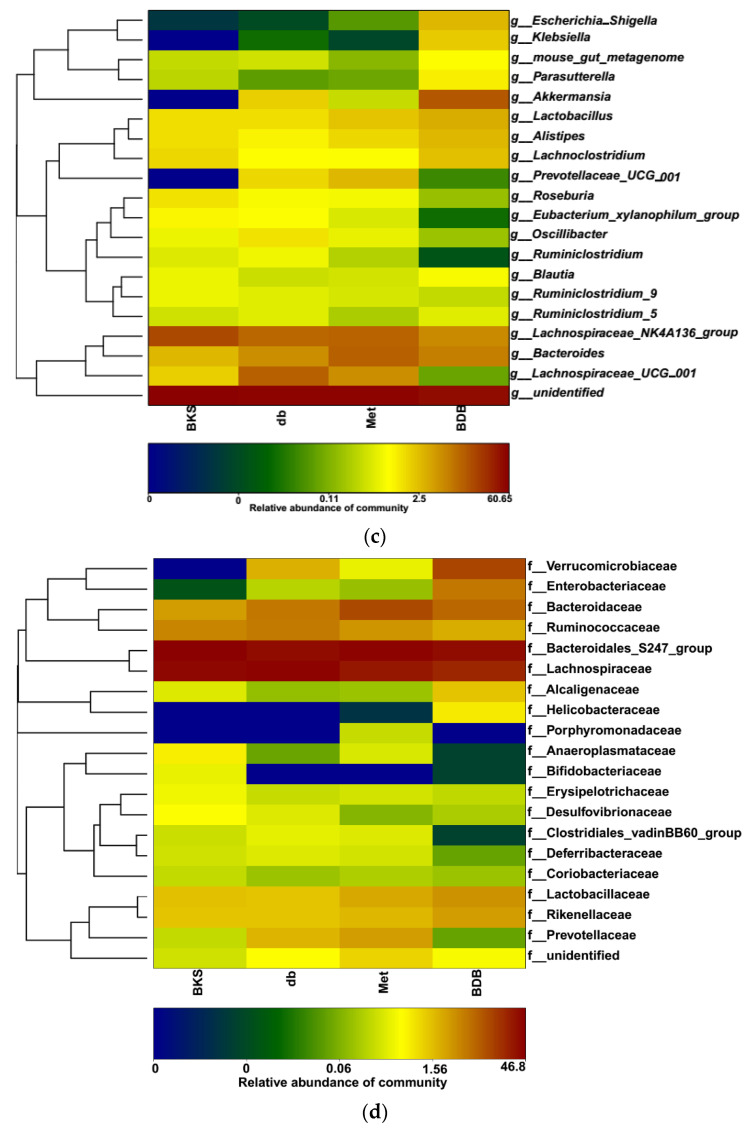
Composition of gut microbiota modulated by BDB and metformin treatment (**a**) Abundance of the representative phyla; (**b**) F/B (Firmicutes to Bacteroidetes) ratio; (**c**,**d**) Heatmap showing the abundance of top 20 bacterial strain at family and genus level. The color from blue to red revealed an abundance of promoting communities. * *p* < 0.05; NS-not significant; db, diabetic BKS db mice; BDB, BDB-treated diabetic mice; Met, metformin-treated group.

We found BDB- and metformin-treatment would affect the overall composition of the gut microbiota. According to our data, the most abundant phyla identified included Bacteroidetes and Firmicutes, which consistist with other resports [22]. The other abundant phyla included *Verrucomicrobia*, *Proteobacteria*, *Actinobacteria*, *Tenericutes*, *Deferribacteres*, *Cyanobacteria* and several unclassified bacteria (Figure 3a). Compared with the group of BKS db mice, metformin treatment significantly increased the relative abundance of Bacteroidetes (*p* < 0.05), while BDB treatment significantly increased the phyla of *Verrucomicrobia* (*p* < 0.001). The relative abundance of Firmicutes decreased significantly in the BDB-treated group (*p* < 0.05). Besides, the ratio of Firmicutes to Bacteroidetes (F/B) decreased in both metformin- and BDB-treatment groups (Figure 3b). The ratio of Firmicutes to Bacteroidetes could be used as an indicator of imbalance of gut microbiota in diabetic and obese mice [23,24]. With that, we analyzed the fecal samples at the family level, which showed dramatically increasing bacteria in the BDB-treated group, such as *Verrucomicrobiaceae*, compared with the BKS db group (*p* < 0.001). The relative abundance of Bacteroidales_S24-7_group showed a similar trend after the BDB treatment. Meanwhile, *Eubacteriaceae*, *Lactobacillaceae* and *Rikenellaceae* showed less abundance after 7-week BDB intervention (Figure 3c). At a deeper bacterial taxa level, a greater detailed difference was observed (Figure 3d). The heatmap showed the bacteria at the genus level in different samples, *Akkermansia*, Lactobacillus, *Alistipes*, and *Lachnoclostridium* were increased in the BDB-treated group. Meanwhile, the relative abundance of Lachnospiraceae_NK4A136_group, Lachnospiraceae_UCG-001, Prevotellaceae_UCG-001 (*p* < 0.05) decreased sharply. Thus, these results had given overall profiles of gut microbiota in different treated groups, which suggested that the intake of BDB and metformin may benefit the stability of certain gut microbiota and regulate microbial imbalance.

**Figure 4:**
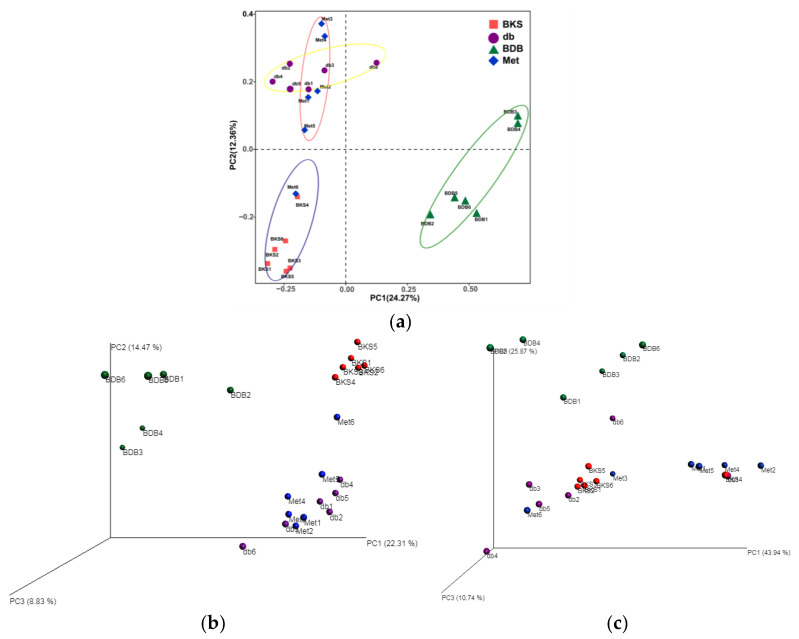
Overall structural modulates of gut microbiota by beta diversity metric among different groups. (**a**) Two-dimensional plot of principal component analysis (PCA); (**b**) Three-dimensional plot of principal coordinates analysis (PCoA) based on weighted UniFrac distances; (**c**) Three-dimensional plot of PCoA based on unweighted UniFrac distances. db, diabetic BKS db mice; BDB, BDB-treated diabetic mice; Met, metformin-treated group.

The beta diversity metric was used to measure the extent of the similarity between microbial communities. Principal component analysis (PCA) and principal coordinates analysis (PCoA) analyses were used in the present study to observe interindividual variation and the microbiota separation. The PCA two-dimensional plot showed that the samples of the BDB-treatment groups were far from the other three groups, and basically gathered on the right side of the vertical axis, while samples of the BKS group were gathered on the left side of the vertical axis (Figure 4a), indicating BDB could drive gut microbiota change along PC2. The BKS db and metformin group had better grouping level on the horizontal axis and separated to some extent. The most significant gut microbial variations were induced by BDB which formed a separate cluster compared with other groups. The result also showed that the total diversity captured by the top two principal coordinates was 36.6%. Analysis of similarity (ANOSIM) statistical analysis of OTUs number of mice in each group showed that there were more remarkable differences in community structure between groups (*p* = 0.001, R = 0.6761), which indicates that the difference between groups is greater than that within groups, and the experimental group is meaningful. The R-value was greater than 0 between groups. The group composition of different samples was further studied based on unweighted (Figure 4b) and weighted ((Figure 4c) UniFrac PCoA analysis. The results showed that segregation degrees between weighted UniFrac PCoA analysis and the unweighted one were different, which revealed that the gut microbiota structure changed significantly by BDB treatment.

### 2.3. Changed Composition of Gut Microbiota

**Figure 5:**
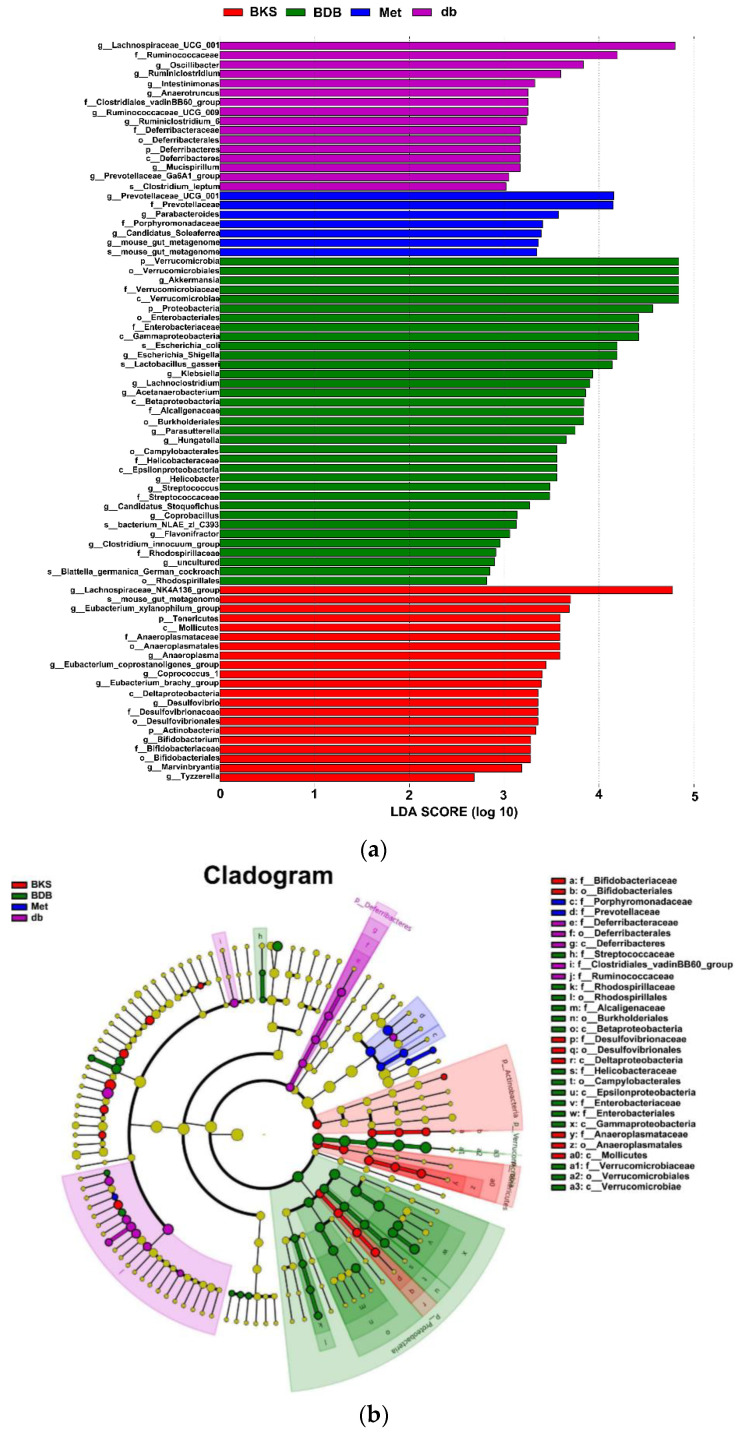
The species significantly changed among normal control (BKS group, red), vehicle-treated diabetic group (db group, purple), BDB-treated group (BDB group, green) and metformin-treated group (Met group, blue). (**a**) Microbial populations by Linear Discriminant Analysis (LDA) scores indicating statistical differences in different groups; (**b**) Evolutionary branch for the special biomarkers of gut microbiota in different groups. db, diabetic BKS db mice; BDB, BDB-treated diabetic mice; Met, metformin-treated diabetic mice.

The differentially abundant taxon was identified by the Linear Discriminant Analysis (LDA) Effect Size (LEfSe) method using non-parametric factorial Kruskal–Wallis and Wilcoxon rank-sum test (*p* < 0.05). The histogram of the distribution of LDA values (LDA > 2.5) shows the statistically different biomarkers between the groups (*p* < 0.05) (Figure 5a). The evolutionary branch diagram shows the differential biomarkers from the phylum to the genus (Figure 5b). The BDB-treated group showed selective enrichment in 35 biomarkers, including *Verrucomicrobia*, *Verrucomicrobiaceae*, *Verrucomicrobiales*, and *Akkermansia*. The metformin-treated group showed a particular effect on *Prevotellaceae* UCG-001 and *Prevotellaceae*, Parabacteroides. Our results indicated that the gut bacteria dysbiosis could be extensively alleviated by metformin and BDB intervention, and certain genera belonging to the 3 predominant phyla may be used as identification biomarkers.

### 2.4. Metagenomic Analysis Revealed Different Functional Profiles between BDB-Treated and Diabetic Model Mice

**Figure 6:**
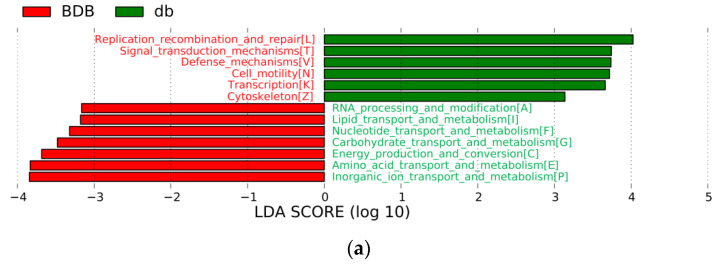

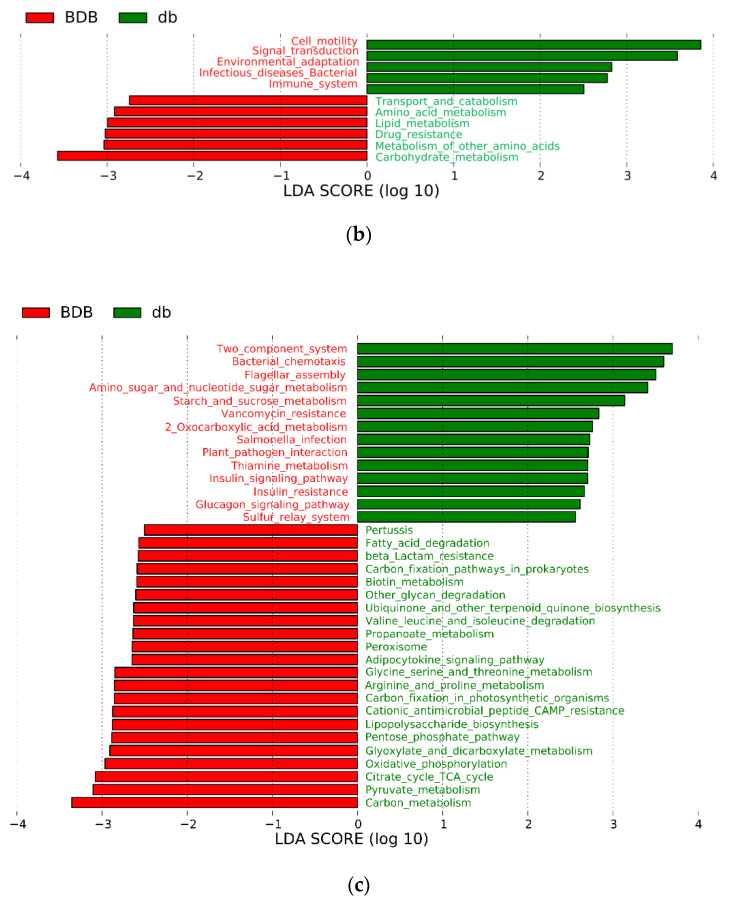
The function of microbial communities comparisons based on Cluster of Orthologous Groups of proteins(COG) category and Kyoto Encyclopedia of Genes and Genomes(KEGG) pathways by LDA Effect Size (LEfSe) analysis. (**a**) The difference of COG category between the BDB and db groups; (**b**,**c**) Histogram of the LDA scores (LDA > 2.5, *p* < 0.05) for differently abundant KEGG pathway at different levels (**b**) At level 2 of KEGG pathway; (**c**) At level 3 of KEGG pathway. BDB, BDB-treated diabetic mice; db, diabetic BKS db mice.

To investigate the effect of BDB on gut microbial function and metabolic pathway in diabetic BKS db mice, we extracted DNA from the feces of mice for metagenomic analysis. We obtained a total of 133,948,850 clean reads (40.2 Gb) and 754,022 open reading frames (ORFs). The Cluster of Orthologous Groups of proteins(COG) and Kyoto Encyclopedia of Genes and Genomes(KEGG) databases were used to annotate the processed data. LEfSe (LDA Effect Size) analysis was used to detect the functional COG categories with significantly different abundances between the BDB-treated mice and diabetic model mice. As shown in Figure 6a, 13 functional COG categories were observed with significantly overabundant reads in the BDB-treated group, which were assigned to the categories of RNA processing and modification (A), Nucleotide transport and metabolism (F), and Lipid transport and metabolism (I), etc. By contrast, the BKS db group had more reads involved in the Cytoskeleton (Z), Transcription (K), Cell motility (N), Defense mechanisms (V), Signal transduction mechanisms (T) and Replication recombination and repair (L) categories. Together, information storage and processing (cluster I) and cellular processes and signaling (cluster II) were summarized in diabetic model mice, and metabolism cluster (cluster III) was predominant in BDB-treated mice.

Furthermore, the KEGG pathway database was used to analyze changes in pathway function between BDB-treated mice and BKS db mice. The significant differences in the KEGG pathway were analyzed by LEfSe. Using the threshold values of LDA > 2.5 and *p* < 0.05, we found that, at level 2, the functional categories related to carbohydrate metabolism, metabolism of other amino acids, drug resistance, lipid metabolism, amino acid metabolism, and transport and catabolism were enriched in the fecal microbiota of BDB-treated mice (Figure 6b), these pathways are generally considered to be important determinants of gut microbiota composition and health [25]. At level 3, 23 KEGG pathways (including citrate cycle, carbon metabolism, valine-leucine-isoleucine degradation, and pyruvate metabolism, and others) and 15 KEGG pathways (including two-component system, glucagon signaling pathway, and bacterial chemotaxis, etc.) were significantly enriched between BDB-treated mice and BKS db mice, respectively (Figure 6c). Compared with BDB-treated group, we found the motility ability of bacteria increased in db/db group (based on the pathway of bacteria motility and flagellar assembly increased significantly). In addition, BDB may alleviate the metabolic disorder of diabetic mice by inhibiting the starch and sucrose metabolism, amino sugar and nucleotide sugar metabolism. Interestingly, multiple representative functional pathways in BDB-treated mice were also involved in metabolism. According to the database of KEGG, the significantly different functional pathways were similar to that in COG categories. Briefly, genes related to the basic metabolism were enriched in the BDB-treated group compared with the BKS db group.

### 2.5. Effects of BDB on Microbial Growth

**Figure 7:**
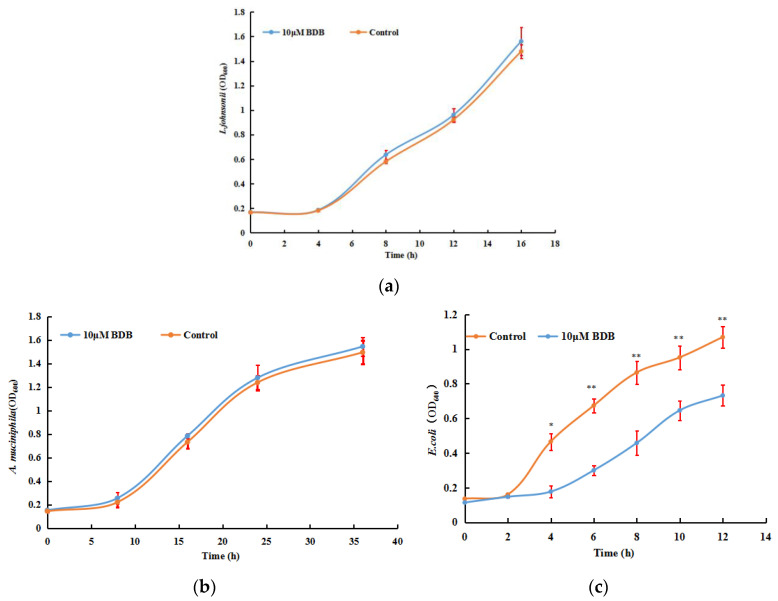
Effects of BDB on the growth of gut bacteria in vitro. (**a**–**c**) *L. johnsonii*, *A. muciniphila*, and *E. coli* as pure cultures in the presence or absence of 10 μM BDB (six technical replicates). * *p* < 0.05, ** *p* < 0.001.

BDB can promote the abundance of short-chain fatty acid-producing bacteria in vivo, which was mentioned earlier in the article. In order to explore whether BDB can directly affect the intestinal flora and its impact on the growth of gut microbiota, we selected significantly increased key communities by BDB to test the interaction between drugs and bacteria in vitro, including *A. muciniphila* and *L. johnsonii*. *E. coli*, which is one of the most common opportunistic pathogens in the intestinal tract and can damage the intestinal mucosal barrier, was also examined. The result showed that BDB tended to promote the growth of *A. muciniphila* and *L. johnsonii* and inhibit the growth of *E. coli*, which indicates the role of promoting and inhibiting proliferation for some special bacterial species (Figure 7a–c). However, the effect of BDB on the growth of *A. muciniphila* and *L. johnsonii* is not significant, which may be due to in vitro culture cannot simulate the environmental conditions of the intestinal tract in vivo. The effects of BDB were probably taken by the inhibition of potential pathogenic/harmful bacteria. BDB-sensitive cells decrease, the proportion of BDB-resistant species, such as *A. muciniphila* and *L. johnsonii* increase.

## 3. Discussion

Currently, the estimated number of uncultured gut microbiota is about 1800, and the microorganisms of the intestinal tract are approximately 10 times that of cells [26]. Therefore, it is difficult to reflect the real gut microbiota status through traditional microbiological techniques, such as the pure culture technique. In recent years, the rapid development of high-throughput sequencing technology provides a new way to better understand the gut microbiota. T2DM is a complex of metabolic disorders, including lipid, protein, and carbohydrate metabolic abnormalities [2]. Previous and present studies demonstrated that 7-week oral administration of BDB significantly decreased the blood glucose levels, suggesting an anti-diabetes effect. In the present study, we explored the gut microbiota composition and function in BDB-treated diabetic mice using both 16S rRNA sequencing and metagenomics sequencing from fecal samples.

The current study showed that BDB treatment for 7 weeks changed the gut microbiota. This was the first time using next-generation sequencing technology to study the effects of BDB on gut microbiota in T2DM in vivo. We observed a change in the alpha diversity from the Chao 1 and observed species. We found that the biodiversity of gut microbiota in the BDB-treatment group decreased significantly compared with the BKS db group, while the metformin-treatment group decreased slightly. Although rich biodiversity may contribute in part to the intestinal homeostasis, the direct modulating effects of specific bacteria may be more critical on host health. Zhang et al. showed berberine-treated hight-fat diet(HFD)-fed mice could effectively relieve insulin resistance and alleviate type 2 diabetes while significantly reducing the gut microbial diversity [27].

A previous study has shown that the dwindling of diversity was closely related to the declining of Firmicutes diversity [28]. We found that F/B decreased in our study, which was consistent with the study of metformin in T2DM mice. We also found that the relative abundance of Firmicutes decreased while some genera, such as *Lactobacilli* related to the production of short chain fatty acids, were increased.

There is no consensus on the effect of anti-diabetic drugs on the ratio of Firmicutes to Bacteroidetes. Sitagliptin has been reported to induce the relative abundance of Bacteroidetes and decrease the relative abundance of Firmicutes [29]. However, saxagliptin, a DPP-4i inhibitor, had the opposite effect, which increased the Firmicutes/Bacteroidetes ratio [30]. Therefore, the beneficial effects of hypoglycemic components might be mediated by specific taxa. This also illustrated that it is too simplistic to reflect the key communities of microbiota at the phylum level. 16S rRNA sequencing combined with metagenomic sequencing is becoming a research hotspot to obtain important variations at the genus levels, gene function and metabolic pathways.

In our present study, some key communities of gut microbiota had high relative abundance when BDB or metformin was supplemented; Metformin improves the growth of the gut bacteria, especially species that produce SCFAs, including Bacteroides and Lactobacillus. Excitedly, large SCFAs-producing species, such as A. muciniphila, Blautia, and Lactobacillus, were enriched after BDB supplementation. Recently, the regulatory effect of drugs and probiotics on gut microbiota in metabolic diseases has been confirmed [31,32]. HFD-induced obese mice with metformin treatment could elevate the Akkermansia spp. to enhance the glucose homeostasis [31]. Also, many studies have shown that SCFAs play an important role in regulating glucose homeostasis [33,34,35]. As the microbial metabolites, SCFAs are associated with health and disease. SCFAs are not only an energy source for intestinal epithelial cells but also signaling molecules for the host [36]. SCFAs can promote cell differentiation and activate G protein-coupled receptor (GPR) 43, which enhances gut barrier function and improve glucose and lipid metabolisms [37]. Interestingly, *A. muciniphila* was not found in BKS mice, but slightly increased in diabetic mice. However, the content of A. muciniphila in BDB-treated group increased by 6-fold (2% vs. 13.7%). *A. muciniphila* could regulate the thickness of the mucus layer, improve the gut barrier function by enhancing the expression of occluding and tight junction protein-1 [38]. *A. muciniphila* was also reported to induce the production of acetate and propionate [13]. It can be speculated that the anti-diabetic effect of BDB may be due to the stimulation of *A. muciniphila*. On the contrary, harmful bacteria, such as Ocillibacter, Lachnospiraceae_NK4A136_group, decreased significantly in BDB- and metformin-treated mice compared with diabetic BKS db mice. Altogether, the accumulation of specific short-chain fatty acid-producing beneficial bacteria is involved in the role of BDB in improving diabetes. The next step is to use metagenomics to examine function changes of gut microbiome after BDB treatment in vivo. In conclusion, we believe that bacterial competition and extracellular compounds play an important role in the anti-diabetic effects of BDB.

Current pieces of evidence indicate that diabetes is closely related to intestinal microbial disorders. But there is no conclusive evidence of what specific microorganisms cause this. Therefore, extensive meta-analysis and studies may benefit us to obtain the key microorganisms, including the beneficial and harmful species, and to obtain important variations at the gene function and metabolic pathway. Metagenomic sequencing was able to overcome the defect of 16S rRNA, especially the poor amplification of some gut bacteria (*Bifidobacteria*, etc.) [39]. It enables functional features analysis of the gut microbiota between diabetic model mice and BDB-treated mice. The COG annotation revealed a significant increase in metabolic pathways in the gut microbiota of BDB-treated mice.

The metabolic pathway or function analysis with the KEGG indicated that gut microbiota function in diabetic mice is closely related to dysregulation of basic metabolic pathways, such as lipid and amino acid metabolism. All the genes were aligned to the KEGG database and the COG database indicated that alterations of the gut microbiota might improve the symptoms of diabetes through metabolic pathways. We have also observed the promotion of basic metabolism (including carbohydrate metabolism and citrate cycle) in the BDB-treated group. We found that the pathogenesis of bacteria (including cell motility and flagellar assembly) was enriched in the BKS db group. We found that propanoate metabolism obviously increased in the BDB treatment mice by KEGG pathway analysis. A previous study showed that metformin treatment increased metabolic activities of lipopolysaccharide biosynthesis, propanoate metabolism, glyoxylate, and dicarboxylate metabolism, and succinate produced by micro bacteria could indirectly improve glucose metabolism through the intestinal gluconeogenesis pathway in mice [34]. These results are consistent with our study. Therefore, fatty acid metabolism may be correlated well with various metabolism diseases [40]. Thus, we suggested that the improvement of diabetic mice treated with BDB was related to these metabolic pathways closely.

In conclusion, our study demonstrated that the mechanism of anti-diabetic effects of BDB is related to changes in the gut microbiota. Although the specific intestinal bacteria altered by BDB and metformin treatment in diabetic mice were significantly different, there was a partial similarity that both showed enrichment of short-chain fatty acid-producing bacteria. Enrichment of short-chain fatty acid-producing bacteria reveals a potential mechanism of BDB for the treatment of diabetes. In brief, the functional difference of microbiota among the BKS db group and the BDB-treated group may indicate that BDB could modulate the dysregulated metabolism pathways of diabetic mice, restoring the normal function of the microbiome.

There are some limitations to our study. Firstly, the number of samples was relatively small. Secondly, we only used the 16S rRNA and macro genomics sequencing method to analyze these preliminary findings. Metabolomics and transcriptome data should be used to further explore these preliminary findings. These techniques are very important to find out the potential interactions between the BDB and gut microbiota and contemplate how gut microbiota can contribute to drugs’ efficacy in clinical practice. Thirdly, our sampling method was limited to feces, which leaves a large part of gut microbiota understudied.

Still, this study provides new evidence for changes in the composition and function of gut microbiota between diabetic model mice and BDB-treated mice and reveals known changes in gut microbiota associated with diabetes. In addition, these interfered gut bacteria are closely related to substantial changes in multiple metabolic pathways. The metabolic potential of the gut microbiota is recognized as popular targets to improve glycemic control of T2DM [41]. Also, our study may provide new mechanisms and insights into the role of gut microbiota in the development and progression of diabetes, and promote novel precision-medicine approaches against T2DM. This study also showed that the analysis of functions and metabolic pathways is more meaningful for the role of gut microbiota in the pathogenesis of diseases compared with the simple comparison of microbial communities. However, the well-understand interaction between drugs and the microbiome is complex and different. Therefore, we need more deep technology (proteomics, transcriptome and metabolomics) to understand the effects microbial metabolites by BDB, and to detect the key microbiota in the treatment of diabetes. It is of great significance to explore the potential usage of BDB in combination with gut microbiota in the future.

## 4. Materials and Methods

### 4.1. Animal Studies

Male wild type BKS mice (C57BLKS/J, the Jackson Laboratory stock number 000662) and BKS db mice (BKS.Cg^-^Dock7^m+/+^Leprdb/J, the Jackson Lab stock number 000642), aged six- to eight-weeks, were supplied by the Model Animal Research Center of Nanjing University (MARC). The mice were provided with individually ventilated cages in a specific pathogen free (SPF) animal rooms and were maintained temperature (24 ± 2 °C), humidity (60–80%), under 12 h light/dark cycle. All mice were fed sterile water and a normal chow diet ad libitum.

The mice for this study were administered according to the guidelines of protocol HAIFAJIZI-2013-3 (approval date: 9 December 2013) approved by the Institute of Oceanology committees for care and use of laboratory animals. A week after the mice adapted to the environment, all diabetic BKS db mice which accord with the experimental requirements were randomly divided into 3 groups: diabetic model group (db group), metformin-treated group (Met group, 100 mg.kg^−1^.body^−1^.day^−1^), BDB-treated group (BDB group, 100 mg.kg^−1^.body^−1^.day^−1^). Age-matched male wild type BKS mice were used as the normal control (BKS group). After 7 weeks, we measured the body weights, food intake, water intake and fasting glucose levels using an Accu-Chek Performa glucometer (Roche, Mannheim, Germany).

### 4.2. Sample Collection

Fecal samples (*n* = 6) were collected under sterile conditions and snap-frozen in liquid nitrogen for half an hour and kept at −80 °C until used in 16S rRNA and metagenomics analysis.

### 4.3. Fecal DNA Extraction, Pyrosequencing and 16S rRNA Gene Sequencing Analysis

Bacterial genomic DNA extraction using the DNA mini kit (Omega, Norcross, GA, USA) from 200 mg frozen fecal. Then the V3-V4 variable regions of bacterial 16S rRNA gene were PCR-amplified using the primers (338F 5′-ACTCCTACGGGAGGCAGCAG-3′, 806R 5′-GGACTACHVGGGTWTCTAAT-3′) and the gene of 16S rRNA was sequenced using the MiseqPE300 platform (service provided by Allwegene Technology Inc (Beijing, China). Raw Fastq files were quality filtering using Trimmomatic (v0.36), Pear (v0.9.6), Flash (v1.20) and Vsearch (v2.7.1). Vsearch was used to gain operational taxonomic units (OTUs) according to 97% sequence similarity and remove the chimeric sequences (2.7.1 https://github.com/torognes/vsearch). The taxonomy of OTUs was analyzed by RDP classifier algorithm (http://rdp.cme.msu.edu/) against the Silva (release 123) 16S rRNA database using confidence threshold of 70%. The relative proportion of each OTU was annotated at the Phylum, Class, Order, Family, Genus and Species levels.

### 4.4. Metagenomics Analyses

The metagenomic sequencing method was used to investigate functional changes in the gut microbiota. A total of 6 fecal samples to extract the genomic DNA (3 samples from BDB-treated BKS db mice and others from placebo-treated diabetic mice).

The qualified DNA samples were randomly interrupted with the size of about 270 bp by covaris ultrasonic crusher. The metagenomic sequencing was carried out on an Illumina HiSeq sequencing platform (Illumina Inc., San Diego, CA, USA) by Allwegene Technology Inc (Beijing, China). The raw sequence reads with quality scores below 20 accounts for more than 50% of the total read length were trimmed. We filtered the low-quality sequences of raw reads obtained by sequencing, and the subsequent analysis was based on filtered clean reads. For the host contamination of the sample, compared with the host database to filter out the reads that may come from the host. The open reading frames (ORFs) were predicted by MetaGeneMark and removed redundant by using CD-HIT software to obtain the non-redundant-nucl (https://github.com/weizhongli/cdhit). The COG functional categories were annotated by the EggNOG database via Smith-Waterman comparison algorithm. The KEGG pathway annotation was performed using a BLAST search by the KEGG database.

### 4.5. Analysis of the Microbiome Composition

Alpha, beta diversity, principal component analysis (PCA) and principal coordinates analysis (PCoA) phylogenetic distance matrix were conducted by the Quantitative Insights into Microbial Ecology (QIIME). The relative abundance of biomarkers among the mice of different groups and the difference of function analyzed with Linear Discriminant Analysis (LDA) Effect Size (LEfSe) method. Only when the *p*-value is less than 0.05, and an LDA value greater than 2.5 is considered to be significantly rich.

### 4.6. Culture of Gut Bacteria

Lactobacillus johnsonii isolated from faeces of the mice, *Akkermansia muciniphila* (DSM22959) was purchased from the BeNa culture collection and *Escherichia coli* were provided by the first institute of oceanography, SOA. They were inoculated in MRS broth medium (de man rogosa Sharpe; Hopebiol, Qingdao, China), modified brain and heart infusion broth medium containing (in g/liter) yeast extract (5), cellobiose (1), maltose (1), cysteine (0.5), mucin (1)and hemin (0.01), and LB broth medium (Hopebiol, Qingdao, China) respectively at a proportion of 1%, and BDB was added to make the final concentration of 10μM in the anaerobic incubator for culture (37 °C, 5% hydrogen, 5% carbon dioxide and 90% nitrogen). OD_600_ was measured continuously at a certain interval, and its growth curve was analyzed.

### 4.7. Statistics Analysis

All the experimental data were presented as the mean ± standard error of the mean (SEM). One-way analysis of variance (ANOVA) followed by Tukey’s post hoc test was used to evaluate the difference between groups. A *p*-value < 0.05 was considered statistically significant. All statistical analyses were determined by SPSS 22.0.
